# Aseasonal Migration of a Northern Bottlenose Whale Provides Support for the Skin Molt Migration Hypothesis

**DOI:** 10.1002/ece3.70921

**Published:** 2025-01-30

**Authors:** K. J. Lefort, L. Storrie, N. E. Hussey, S. H. Ferguson

**Affiliations:** ^1^ Fisheries and Oceans Canada Northwest Atlantic Fisheries Centre St. John's Newfoundland and Labrador Canada; ^2^ Department of Environment and Geography University of Manitoba Winnipeg Manitoba Canada; ^3^ Department of Integrative Biology University of Windsor Windsor Ontario Canada; ^4^ Department of Biological Sciences University of Manitoba Winnipeg Manitoba Canada; ^5^ Fisheries and Oceans Canada Freshwater Institute Winnipeg Manitoba Canada

**Keywords:** cetacean, *Hyperoodon ampullatus*, migration, northern bottlenose whale, skin molt, telemetry

## Abstract

Why animals migrate is a fundamental question in biology. While the adaptive significance of some animal migrations is well understood (e.g., to find food, to pursue more‐favorable habitats, to spawn, or to give birth), others remain unknown. The adaptive significance of whale migration, for example, is unresolved and multiple hypotheses have been proposed to explain it. One recently proposed hypothesis that challenges the long‐standing “feeding‐breeding” whale migration model is a “feeding‐molting” model, where whales undertake latitudinal migrations to warmer waters to molt skin. In July 2019, we attached satellite‐tracking tags to northern bottlenose whales ( *Hyperoodon ampullatus* ) in the Canadian Arctic. One of these tagged whales completed a round‐trip movement between the Arctic and the temperate western North Atlantic, traveling 7281 km in 67 days (and spanning 27° of latitude). The whale was tagged in sea‐surface temperatures of ~4°C, but migrated south, reaching ~23°C surface waters, where it remained for 7 days before returning to the Arctic. The whale's occupancy of warm water was accompanied by a distinct shift in dive behavior, remaining near the ocean's surface. Four other tagged whales initiated similar long‐distance movements. We conclude that feeding or breeding were unlikely reasons for this movement and that northern bottlenose whales migrate to warmer latitudes to molt skin.

## Graphical abstract

We attached satellite‐tracking tags to northern bottlenose whales ( *Hyperoodon ampullatus* ) in the Canadian Arctic to explore the species' movement ecology. One of the tagged whales completed a round‐trip movement between the Arctic and the temperate western North Atlantic, traveling 7281 km in 67 days. Our data suggest that feeding or breeding were unlikely reasons for this movement, and we propose instead that northern bottlenose whales migrate to lower latitudes to molt skin.

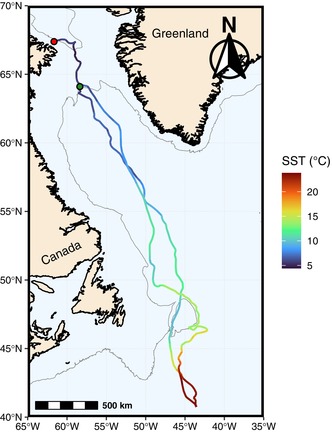

## 1 Introduction

Why animals migrate^1^ is a fundamental question in biology (see Dingle 1996). Recent advances in animal tracking technology (e.g., smaller electronic tracking devices and longer battery life) have allowed biologists to document unprecedented animal migrations (e.g., Croxall et al. 2005; Egevang et al. 2010; Hussey et al. 2015). Yet despite these technological advances, the question of why animals migrate remains unanswered for many species. Mysticete (baleen) and odontocete (toothed) whales, for example, undertake some of the longest‐distance movements among mammals, often traveling thousands of kilometers between the areas that they occupy at different times of the year (Hucke‐Gaete et al. 2018; Lefort et al. 2022; Lydersen et al. 2020; Matthews et al. 2011; Stone, Florez‐Gonzalez, and Katona 1990). For example, gray whales ( *Eschrichtius robustus* ) migrate between the Pacific subarctic (where they spend the summer) and the subtropical eastern North Pacific (where they spend the winter). In fact, a satellite‐tracked gray whale holds the record for the longest‐documented migration among all mammals: a 22,000+ km round‐trip journey between the Sea of Okhotsk, Russia, and near the Baja California peninsula, Mexico (Mate et al. 2015). However, there is uncertainty about the adaptive significance of these migrations, and multiple hypotheses have been proposed to explain them (Corkeron and Connor 1999; Pitman et al. 2020). Ultimately, why whales migrate remains unresolved.

Many mysticetes undertake seasonal migrations between high and low latitudes (Stern and Friedlaender 2018), thereby occupying an arena that undergoes seasonal changes in light, temperature, and productivity (MacArthur 1972). Indeed, some authors have suggested that when the spatial distribution of resources fluctuates seasonally, migration should predominate over residency (Avgar, Street, and Fryxell 2014; Fryxell and Holt 2013). The prevailing hypothesis explaining mysticete migration, the “feeding‐breeding” model (i.e., seasonal movements between high‐latitude summer feeding grounds and low‐latitude winter breeding grounds) appears to agree, at least partially, with seasonal variation in productivity. There is consensus that whales benefit from feeding at high latitudes during summer when food is abundant; however, the advantage(s) of migrating to low latitudes in the winter, where food is scarce, for breeding remains the subject of debate (Clapham 2001; Corkeron and Connor 1999; Pitman et al. 2020). That said, there are many examples of mysticete migratory movements and their seasonal partitioning of behaviors (i.e., feeding and breeding) that seem to provide correlational support for this model (Stern and Friedlaender 2018). Odontocete movements are more varied and complex than those of mysticetes, with some species exhibiting seemingly migratory patterns (e.g., Lefort et al. 2022; Matthews et al. 2011), while others exhibit patterns of residency or nomadism (e.g., Bigg et al. 1990; Herzing et al. 2017; Servidio et al. 2019). While the feeding‐breeding model is generally applied to mysticetes, there are examples of odontocetes undertaking similar long‐distance movements from high to low latitudes in autumn (e.g., Lefort et al. 2022; Matthews et al. 2011).

Recently, the feeding‐breeding model was challenged by Pitman et al. (2020) when the authors tracked rapid long‐distance (up to 11,000 km round‐trip) return migrations (with little time spent at the turnaround point) by four different ecotypes of killer whales ( *Orcinus orca* ) between the coastal waters of Antarctica and the subtropics (Durban and Pitman 2012; Pitman et al. 2020). In contrast to the seasonal feeding‐breeding model, Pitman et al. (2020) proposed a “feeding‐molting” model explaining these seemingly aseasonal^2^ movements. That is, that Antarctic killer whales undertake “skin molt migrations”, periodically moving into warmer waters to molt skin, before returning to cold productive Antarctic waters to continue feeding. This proposition is based on the fact that whales must increase blood flow to the skin's surface to molt, but this can be a challenge in cold waters where whales must restrict blood flow to the skin's surface to limit heat loss. Durban and Pitman (2012) and Pitman et al. (2020) cite diatom accumulation on the skin of killer whales in Antarctic waters as evidence that normal skin molt is not occurring in this area. Molting may be essential for cetaceans to maintain a healthy skin biome (Hooper et al. 2019; Van Cise et al. 2020). So, the movements documented by Pitman et al. (2020) may allow killer whales to molt periodically to maintain healthy skin (e.g., removing potential pathogens from the skin), while still allowing them to forage in physiologically challenging, but productive Antarctic waters. Furthermore, Pitman et al. (2020) proposed skin molt as a driver for all high‐low latitude whale migrations. To our knowledge, since the publication of Pitman et al.'s (2020) seminal work, there has been no additional published support for the idea of skin molt migration among cetaceans.

The northern bottlenose whale ( *Hyperoodon ampullatus* ) is a deep‐diving, primarily squid‐eating odontocete endemic to the deep waters of the North Atlantic Ocean. The species is listed as Near Threatened by the International Union for Conservation of Nature (Whitehead et al. 2021). Our understanding of the species' ecology has grown considerably since the 1990s (e.g., Feyrer et al. 2024; Gowans et al. 2000; de Greef et al. 2022; Whitehead et al. 1997); despite this, the species' migratory patterns, if any exist, remain largely unknown. That said, northern bottlenose whales are seasonal visitors to the Azores Archipelago (Silva et al. 2013) and have also been observed as far south as the Canary Islands (Simmonds and Lopez‐Jurado 1991); this provides some support for seasonal movements to low latitudes, at least in the eastern North Atlantic (although see Whitehead and Hooker 2012). Here, we describe a satellite‐tracked aseasonal round‐trip migration by a northern bottlenose whale between the Canadian Arctic and the temperate western North Atlantic. The southern terminus of the migration was characterized by surface water ~19°C warmer than where the whale was tagged, and by a distinct shift in dive behavior where the animal remained near the ocean's surface. We propose that, like Antarctic killer whales, northern bottlenose whales migrate to warmer latitudes not to feed or breed, but to molt skin.

## 2 Methods

**FIGURE 1:**
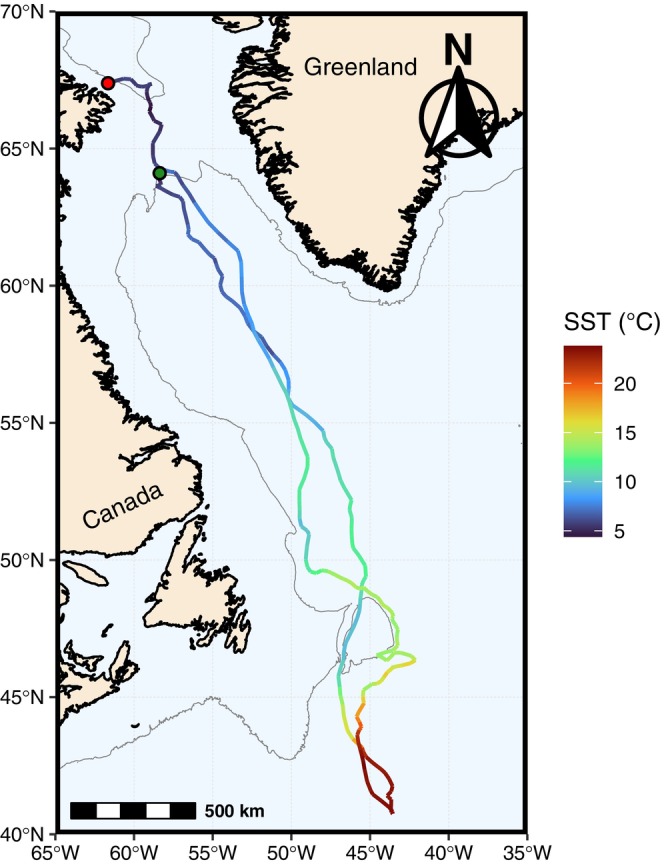
Model‐estimated path of an adult male northern bottlenose whale ( *Hyperoodon ampullatus* ) satellite tracked along the eastern Canadian coast from July 5 to September 10, 2019. The green circle is the tagging location and the red circle is the final model‐estimated location. The thin gray line is the 1000 m depth contour (data obtained from the ETOPO 2022 Global Relief Model using the *marmap* R package; Pante, Simon‐Bouhet, and Irisson 2023). The track is color‐coded by the mean daily sea‐surface temperature (°C) along the whale's migration.

In July 2019, we attached satellite‐linked data transmitters (Cetacean LIMPET SPOT5 and SPLASH10, Wildlife Computers; hereafter, tags) to northern bottlenose whales in Davis Strait (tagging location: 64.11° N, 58.37° W; Figure 1). Using a small inflatable boat, we approached adult northern bottlenose whales near a commercial gillnet‐fishing vessel, where they were observed in close proximity. We deployed tags using a 150‐lb draw‐weight crossbow, securing tags with two titanium anchors into epidermal‐dermal tissue near the base of the dorsal fin. We programmed tags to transmit every day during July–September. We also programmed SPLASH10 tags to record depth and temperature for 1‐h bouts at 75‐s intervals (hereafter, tag‐recorded depth and tag‐recorded temperature).

To explore the possibility that a long‐distance movement completed by one of the tagged whales was motivated by a need to molt, we grouped that whale's data into three hypothetical phases based on the animal's movements and the temperatures it experienced: foraging phase, directed‐movement phase, and molting phase. The foraging phase was defined by latitudes above 63.5° N (July 5–8 and September 2–10), near where the animal was tagged. This phase was characterized by tortuous movements (i.e., slower movements with more directional changes), indicative of foraging. The molting phase was defined by the period at the whale's southern terminus where it encountered a sharp increase in sea‐surface temperature (SST) and remained in those higher SSTs for 7 days (July 30–August 5). The SSTs observed during this period (i.e., > 22.0°C) agreed with the northern terminus temperatures observed by Pitman et al. (2020), so we selected 22.0°C as the threshold to delineate this phase from the directed‐movement phase. The directed‐movement phase was defined as the periods between the foraging and molting phases (July 9–29 and August 6–September 1). This phase was characterized by directionally persistent movements.

We fit a correlated random walk model to raw location data that accounted for satellite‐derived location accuracy to estimate locations at 6‐h intervals using the *aniMotum* R package (Jonsen et al. 2023). We calculated distance (km) between successive model‐estimated locations using the *geosphere* R package (Hijmans 2022) and calculated daily distance traveled (km day^−1^) by summing the distances between all successive model‐estimated locations each day. We obtained SST at each model‐estimated location from the ¼° Daily Optimum Interpolation Sea Surface Temperature (OISST) database (Huang et al. 2020). We downloaded daily SST layers for each day the tag transmitted and extracted SST from the cell that each model‐estimated location fell within from the layer corresponding to the day that model‐estimated location was generated. We present SST as a daily mean (i.e., the mean SST the whale experienced that day). We present tag‐recorded depth data (dive data) as a mean of the daily maximums during each of the three hypothetical phases. We present tag‐recorded temperature data as a mean of daily means during each of the three hypothetical phases. Finally, we compared temperature and depth metrics among the three hypothetical phases. We completed all data handling in R (R Core Team 2023) using the *tidyverse* collection of R packages (Wickham et al. 2019). Summary data are presented as means ± standard deviations.

## 3 Results

**FIGURE 2:**
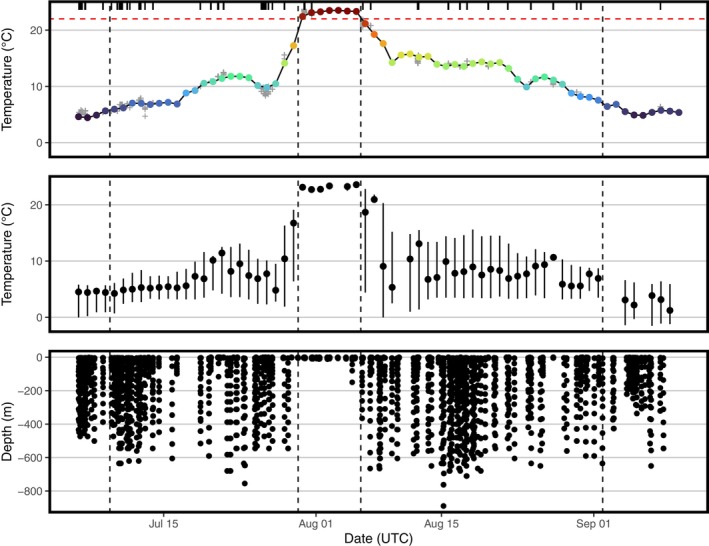
Top: Mean daily sea‐surface temperature (°C; color‐coded by temperature to match Figure 1) along the northern bottlenose whale's ( *Hyperoodon ampullatus* ) migration (obtained from the OISST database). Gray crosses show tag‐recorded temperature data when at the ocean's surface (< 5 m depth), demonstrating agreement between tag‐recorded temperature and the OISST. Rug plot shows the temporal distribution of tag‐recorded temperature data when at the ocean's surface. Horizontal red dashed line shows 22.0°C, the temperature threshold used to define the molting phase. Vertical dashed lines show the breaks between the three hypothetical movement phases: foraging phase (July 5–8 and September 2–10), directed‐movement phase (July 9–29 and August 6–September 1), and molting phase (July 30–August 5). Middle: Tag‐recorded temperature (°C) along the whale's migration. Points show daily means. Vertical bars show daily minima and maxima. Bottom: Tag‐recorded depth (m) along the whale's migration.

We identified the tagged animal as an adult male based on the shape, size, and color of its melon (which is enlarged, flattened, and generally lighter in color in mature males). The tagged whale completed a rapid round‐trip movement between the Canadian Arctic and the temperate western North Atlantic, traveling 7281 km in 67 days (July 5–September 10) (Figure 1; also see Figure S1). Daily distanced traveled was 108.7 ± 44.1 km day^−1^ (range: 25.5–217.2 km day^−1^) (Figure S2). The tag transmitted 3.8 ± 2.9 h of tag‐recorded depth data and 3.9 ± 2.8 h of tag‐recorded temperature data each day. Mean daily SST ranged from 4.4°C to 23.8°C (Figure 2). Tag‐recorded temperature ranged from −1.5°C to 23.7°C. The maximum tag‐recorded depth was 890 m. The whale's southernmost model‐estimated location was 40.75° N, 43.56° W (Figure 1).

SSTs were coldest during the foraging phase (5.4°C ± 0.7°C; tag‐recorded temperature = 3.5°C ± 1.2°C) followed by the directed‐movement phase (11.6°C ± 3.6°C; tag‐recorded temperature = 8.2°C ± 3.4°C) (Figure 2). Daily maximum tag‐recorded depths were deep during both the foraging (432.2 ± 124.7 m) and directed‐movement (532.4 ± 193.3 m) phases. Furthermore, maximum daily depths ≥ 100 m were recorded on 52 of 55 days with available data during the foraging and directed‐movement phases. During the proposed molting phase, the whale occupied warmer SSTs (23.2°C ± 0.4°C; tag‐recorded temperature = 23.1°C ± 0.3°C) and shifted its dive behavior to remain near the ocean's surface (daily maximum tag‐recorded depth = 29.5 ± 53.2 m). This included six consecutive days (July 30–August 4) where the tag‐recorded depth did not exceed 11.5 m.

Finally, in addition to the round‐trip migration described above, four other tagged northern bottlenose whales (of the six that we tagged and for which we had > 3 days of data) initiated similar rapid, southward movements, but unfortunately, their transmissions were not sustained long enough (i.e., tags likely detached prematurely) to document a complete round‐trip migration (Figure S3). These movements were initiated within a few days of each other. The sixth tagged whale did not initiate a southward movement and remained above 63.5° N until the tag stopped transmitting on July 11 (Figure S3).

## 4 Discussion

For decades, the feeding‐breeding paradigm has dominated the narrative as to why whales migrate (Clapham 2001; Corkeron and Connor 1999; Kellogg 1929; Mackintosh 1965). This narrative has principally been inferred through observations of seasonal migrations and the behaviors (feeding and breeding) associated with them (e.g., Corkeron, Ensor, and Matsuoka 1999; Konishi et al. 2024; Szesciorka et al. 2020; Visser et al. 2011). We documented the timing and path of a rapid, long‐distance, round‐trip migration by a northern bottlenose whale, spanning the Canadian Arctic and the temperate western North Atlantic. This migration was aseasonal (i.e., it occurred during the “feeding” season) and was characterized by movement into surface water ~19°C warmer than where the whale was tagged and by a distinct shift in dive behavior at its southern terminus. In contrast to seasonal migrations, which are typically characterized by cetaceans spending several months at lower latitudes (De Weerdt et al. 2023; Risch et al. 2014; Romagosa et al. 2020; Shabangu et al. 2019), the northern bottlenose whale in this study spent only 7 days at its southern terminus.

While we recognize that our study is based on only a single animal (although see Figure S3), we find these data to support a feeding‐molting migration model. We reached this conclusion as our data do not align with other previously hypothesized drivers of whale migration (i.e., feeding, mating, or calving), which we discuss in detail below. Also noteworthy is the fact that comparable round‐trip movements (i.e., rapid south–north movements with little time spent at the turnaround point) by satellite‐tracked northern bottlenose whales have also been documented in the eastern North Atlantic, occurring between the Nordic Seas and near the Azores Archipelago (Paulus Wensveen, pers. comm.).

We believe it is unlikely that the round‐trip movement documented here was to feed. Firstly, the Canadian Arctic offers abundant and easily obtainable food resources to northern bottlenose whales during the open‐water season, notably via discards from the commercial fishery. Secondly, the tagged whale, which is a known deep‐water forager (Hooker and Baird 1999), was recorded only reaching a maximum depth of 11.5 m during the suspected molt (with the exception of one dive to 150 m on the last day of the suspected molt). We recognize that we may have “missed” dives, which could bias our results, during gaps in our coverage of the animal's depth profile (Figure S4). However, because there were no indications of temporal patterns in our coverage of the animal's depth profile (Figure S4) or of diel patterns in dive behavior (Figure S5), we can reasonably assume that the likelihood of “missing” a dive did not differ among the three phases. Thus, the presence of deep dives throughout the study, except during the suspected molt (Figure 2), provides strong support for a distinct shift in dive behavior during this time. The lack of deep diving presumably allowed the whale to remain in relatively warm surface waters conducive to molting. This is in contrast to the whale's dive behavior on the Arctic foraging grounds and during its directed‐movement phase, where frequent deep dives (> 500 m) were recorded, perhaps allowing the whale to feed in preparation for and to recover from a week‐long period of fasting during the molt. This behavior during long‐distance movements is arguably inconsistent with migrations as per some definitions of the word (e.g., Kennedy 1985; where maintenance activities, such as feeding, cease during migratory movements), as has been discussed elsewhere (see Storrie et al. 2023). Nonetheless, the 6‐day hiatus in dives beyond 11.5 m at the southern terminus of the whale's movement is strong evidence that this migration was not to feed.

Equally, we believe it is unlikely that this migration was for breeding. We can discount calving as the whale was a male. We also consider it unlikely (although possible) that this migration was motivated by mating. de Greef et al. (2022) described a genetic subpopulation among whales sampled in the Canadian Arctic, Labrador, and Newfoundland, which would suggest that mating occurs among whales from these areas. However, mating among northern bottlenose whales from this population is thought to occur in spring and early summer (Benjaminsen 1972; although also see Whitehead et al. 1997), placing this possible “mating migration” outside of that temporal window.

We agree with Pitman et al. (2020) that molting may be the primary driver of migration for all whales that forage at high latitudes and migrate to tropical waters. As alluded to by Pitman et al. (2020), synchronizing multiple behaviors (e.g., mating, calving, nursing, and molting) at low latitudes may be highly adaptive for seasonal migrants, but these multiple behaviors make it difficult to parse out which has the greatest evolutionary fitness advantage, and is the true driver of such long‐distance movements. Aseasonal migrations, such as those observed in Antarctic killer whales (Durban and Pitman 2012; Pitman et al. 2020) and the northern bottlenose whales in this study, can help to elucidate the primary driver of these movements. That said, many questions concerning whale migrations, especially in elusive species such as northern bottlenose whales, remain unanswered. For example, we do not know if all northern bottlenose whales foraging in Arctic waters complete these aseasonal round‐trip movements or how often they occur. Fish harvesters observe whales in Davis Strait throughout the open‐water season, so it is unknown what cues (e.g., physiological, environmental, or social) trigger these migrations. In light of this, we advocate for continued satellite‐tagging efforts as a means to expand our understanding of whale migrations. As a specific example, if satellite‐tagged pre‐breeders completed similar movements, we could more confidently eliminate breeding as the reason for these movements. These ongoing tagging efforts will contribute to a more comprehensive understanding of whale movements, perhaps eventually allowing us to answer the age‐old question: why do whales migrate?

## Author Contributions

**K. J. Lefort:** conceptualization (equal), data curation (lead), formal analysis (lead), investigation (lead), methodology (lead), software (lead), validation (lead), visualization (lead), writing – original draft (lead), writing – review and editing (lead). **L. Storrie:** conceptualization (equal), data curation (supporting), formal analysis (supporting), investigation (supporting), methodology (supporting), software (supporting), validation (supporting), visualization (supporting), writing – review and editing (equal). **N. E. Hussey:** conceptualization (equal), funding acquisition (equal), project administration (equal), resources (equal), writing – review and editing (equal). **S. H. Ferguson:** conceptualization (equal), funding acquisition (equal), project administration (equal), resources (equal), supervision (lead), writing – review and editing (equal).

## Ethics Statement

Fieldwork was approved by the Fisheries and Oceans Canada (DFO) Freshwater Institute Animal Care Committee under Animal Use Protocol #FWI‐ACC‐2019‐09 and permitted by DFO under License to Fish for Scientific Purposes #S‐19/20‐1006‐NU.

## Conflicts of Interest

The authors declare no conflicts of interest.

## Supporting information


**Data S1:** Locations.

**Data S2:** OISST.

**Data S3:** README.

**Data S4:** Series.

**Data S5:** skin_molt_code.R.

**Figure S1.** Model‐estimated path of an adult male northern bottlenose whale ( *Hyperoodon ampullatus* ) satellite tracked along the eastern Canadian coast from July 5 to September 10, 2019. The green circle is the tagging location and the red circle is the final model‐estimated location. The ocean is color‐coded by sea‐surface temperature (°C) on July 30, 2019 (the first day of the proposed molt) obtained from the OISST database. Isotherms are drawn at 0°C, 10°C, and 20°C (dashed lines).

**Figure S2.** Daily distance traveled (km day^−1^) along the northern bottlenose whale's ( *Hyperoodon ampullatus* ) satellite‐tracked path. Vertical dashed lines show the breaks between the three hypothetical movement phases: foraging phase (July 5–8 and September 2–10), directed‐movement phase (July 9–29 and August 6–September 1), and molting phase (July 30–August 5).

**Figure S3.** Model‐estimated path of five other adult northern bottlenose whales ( *Hyperoodon ampullatus* ) satellite‐tagged in Davis Strait in July 2019. Panel A: four whales which initiated southward movements (but whose transmissions were not sustained long enough to document a complete round‐trip migration, if one did occur). Panel B: one whale which did not initiate a southward movement. Each color represents an individual whale.

**Figure S4.** Top: Histogram showing the number of tag‐recorded depth observations collected each day from a satellite‐tracked northern bottlenose whale ( *Hyperoodon ampullatus* ). Bottom: Diel distribution of tag‐recorded depth observations collected each day. Vertical dashed lines show the breaks between the three hypothetical movement phases: foraging phase (July 5–8 and September 2–10), directed‐movement phase (July 9–29 and August 6–September 1), and molting phase (July 30–August 5).

**Figure S5.** Top: Histogram showing the number of tag‐recorded depth observations binned by hour of day from a satellite‐tracked northern bottlenose whale ( *Hyperoodon ampullatus* ). Bottom: raw tag‐recorded depth (m) by time of day (UTC) across the entire deployment. The whale occupied longitudes between 42° W and 62° W, occupying time zones −3 and −4 UTC; thus, midday local time ranged from 09:00 to 08:00 UTC.

## Data Availability

Data and code are available as Supporting Information.

## Endnotes

^1^
In this article, we define *migration* as *the long‐distance, persistent movement of individual animals between areas*. This conforms to its typical use in the cetacean literature (e.g., Corkeron and Connor 1999; Geijer, Notarbartolo di Sciara, and Panigada 2016; Stern and Friedlaender 2018). However, we recognize that other definitions exist (see Dingle 2006; Kennedy 1985), some of which do not agree with the definition used here (see Storrie et al. 2023 for discussion).

^2^
In this article, we use *aseasonal* to describe migrations that do not fit the *seasonal* feeding‐breeding model (i.e., long‐distance seasonal movements between high‐latitude summer feeding grounds and low‐latitude winter breeding grounds).

## References

[ece370921-bib-0001] Avgar, T. , G. Street , and J. M. Fryxell . 2014. “On the Adaptive Benefits of Mammal Migration.” Canadian Journal of Zoology 92: 481–490. 10.1139/cjz-2013-0076.

[ece370921-bib-0002] Benjaminsen, T. 1972. “On the Biology of the Northern Bottlenose Whale, *Hyperoodon ampullatus* (Forster).” Norwegian Journal of Zoology 20: 233–241.

[ece370921-bib-0003] Bigg, M. A. , P. F. Olesiuk , G. M. Ellis , J. K. Ford , and K. C. Balcomb . 1990. “Social Organization and Genealogy of Resident Killer Whales (*Orcinus orca*) in the Coastal Waters of British Columbia and Washington State.” Report of the International Whaling Commission 12: 383–405.

[ece370921-bib-0004] Clapham, P. 2001. “Why Do Baleen Whales Migrate? A Response to Corkeron and Connor.” Marine Mammal Science 17: 432–436. 10.1111/j.1748-7692.2001.tb01289.x.

[ece370921-bib-0005] Corkeron, P. J. , and R. C. Connor . 1999. “Why Do Baleen Whales Migrate?” Marine Mammal Science 15: 1228–1245. 10.1111/j.1748-7692.1999.tb00887.x.

[ece370921-bib-0006] Corkeron, P. J. , P. Ensor , and K. Matsuoka . 1999. “Observations of Blue Whales Feeding in Antarctic Waters.” Polar Biology 22: 213–215. 10.1007/s003000050412.

[ece370921-bib-0007] Croxall, J. P. , J. R. Silk , R. A. Phillips , V. Afanasyev , and D. R. Briggs . 2005. “Global Circumnavigations: Tracking Year‐Round Ranges of Nonbreeding Albatrosses.” Science 307: 249–250. 10.1126/science.1106042. 15653503

[ece370921-bib-0008] de Greef, E. , A. L. Einfeldt , P. J. Miller , et al. 2022. “Genomics Reveal Population Structure, Evolutionary History, and Signatures of Selection in the Northern Bottlenose Whale, *Hyperoodon ampullatus* .” Molecular Ecology 31: 4919–4931. 10.1111/mec.16643. 35947506 PMC9804413

[ece370921-bib-0009] De Weerdt, J. , A. S. Pacheco , J. Calambokidis , et al. 2023. “Migratory Destinations and Spatial Structuring of Humpback Whales ( *Megaptera novaeangliae* ) Wintering off Nicaragua.” Scientific Reports 13: 15180. 10.1038/s41598-023-41923-7. 37704666 PMC10500005

[ece370921-bib-0010] Dingle, H. 1996. Migration: The Biology of Life on the Move. New York: Oxford University Press.

[ece370921-bib-0011] Dingle, H. 2006. “Animal Migration: Is There a Common Migratory Syndrome?” Journal für Ornithologie 147: 212–220. 10.1007/s10336-005-0052-2.

[ece370921-bib-0012] Durban, J. W. , and R. L. Pitman . 2012. “Antarctic Killer Whales Make Rapid, Round‐Trip Movements to Subtropical Waters: Evidence for Physiological Maintenance Migrations?” Biology Letters 8: 274–277. 10.1098/rsbl.2011.0875. 22031725 PMC3297399

[ece370921-bib-0013] Egevang, C. , I. J. Stenhouse , R. A. Phillips , A. Petersen , J. W. Fox , and J. R. Silk . 2010. “Tracking of Arctic Terns *Sterna paradisaea* Reveals Longest Animal Migration.” Proceedings of the National Academy of Sciences of the United States of America 107: 2078–2081. 10.1073/pnas.0909493107. 20080662 PMC2836663

[ece370921-bib-0014] Feyrer, L. J. , J. E. Stanistreet , C. Gomez , et al. 2024. “Identifying Important Habitat for Northern Bottlenose and Sowerby's Beaked Whales in the Western North Atlantic.” Aquatic Conservation: Marine and Freshwater Ecosystems 34: e4064. 10.1002/aqc.4064.

[ece370921-bib-0015] Fryxell, J. M. , and R. D. Holt . 2013. “Environmental Change and the Evolution of Migration.” Ecology 94: 1274–1279. 10.1890/12-0668.1. 23923490

[ece370921-bib-0016] Geijer, C. K. A. , G. Notarbartolo di Sciara , and S. Panigada . 2016. “Mysticete Migration Revisited: Are Mediterranean Fin Whales an Anomaly?” Mammal Review 46: 284–296. 10.1111/mam.12069.

[ece370921-bib-0017] Gowans, S. , H. Whitehead , J. Arch , and S. K. Hooker . 2000. “Population Size and Residency Patterns of Northern Bottlenose Whales ( *Hyperoodon ampullatus* ) Using the Gully.” Journal of Cetacean Research and Management 2: 201–210. 10.47536/jcrm.v2i3.908.

[ece370921-bib-0018] Herzing, D. L. , B. N. Augliere , C. R. Elliser , M. L. Green , and A. A. Pack . 2017. “Exodus! Large‐Scale Displacement and Social Adjustments of Resident Atlantic Spotted Dolphins ( *Stenella frontalis* ) in The Bahamas.” PLoS One 12: e0180304. 10.1371/journal.pone.0180304. 28792947 PMC5549894

[ece370921-bib-0019] Hijmans, R. J. 2022. “geosphere: Spherical Trigonometry. R package version 1.5‐18.” https://CRAN.R‐project.org/package=geosphere.

[ece370921-bib-0020] Hooker, S. K. , and R. W. Baird . 1999. “Deep‐Diving Behaviour of the Northern Bottlenose Whale, *Hyperoodon ampullatus* (Cetacea: Ziphiidae).” Proceedings of the Royal Society B 266: 671–676. 10.1098/rspb.1999.0688.

[ece370921-bib-0021] Hooper, R. , J. C. Brealey , T. van der Valk , et al. 2019. “Host‐Derived Population Genomics Data Provides Insights Into Bacterial and Diatom Composition of the Killer Whale Skin.” Molecular Ecology 28: 484–502. 10.1111/mec.14860. 30187987 PMC6487819

[ece370921-bib-0022] Huang, B. , C. Liu , V. Banzon , et al. 2020. “Improvements of the Daily Optimum Interpolation Sea Surface Temperature (DOISST) Version 2.1.” Journal of Climate 34: 2923–2939. 10.1175/JCLI-D-20-0166.1.

[ece370921-bib-0023] Hucke‐Gaete, R. , L. Bedriñana‐Romano , F. A. Viddi , J. E. Ruiz , J. P. Torres‐Florez , and A. N. Zerbini . 2018. “From Chilean Patagonia to Galapagos, Ecuador: Novel Insights on Blue Whale Migratory Pathways Along the Eastern South Pacific.” PeerJ 6: e4695. 10.7717/peerj.4695. 29736336 PMC5933318

[ece370921-bib-0024] Hussey, N. E. , S. T. Kessel , K. Aarestrup , et al. 2015. “Aquatic Animal Telemetry: A Panoramic Window Into the Underwater World.” Science 348: 1255642. 10.1126/science.1255642. 26068859

[ece370921-bib-0025] Jonsen, I. D. , W. J. Grecian , L. Phillips , et al. 2023. “aniMotum, an R Package for Animal Movement Data: Rapid Quality Control, Behavioural Estimation and Simulation.” Methods in Ecology and Evolution 14: 806–816. 10.1111/2041-210X.14060.

[ece370921-bib-0026] Kellogg, R. 1929. “What is Known on the Migrations of Some of the Whalebone Whales?” Smithsonian Institution Annual Report. Washington, USA 467–494.

[ece370921-bib-0027] Kennedy, J. S. 1985. “Migration, Behavioral and Ecological.” Migration: Mechanisms and Adaptive Significance 27: 5–26.

[ece370921-bib-0028] Konishi, K. , S. Minamikawa , L. Kleivane , and M. Takahashi . 2024. “Annual Phenology and Migration Routes to Breeding Grounds in Western‐Central North Pacific Sei Whales.” Scientific Reports 14: 11212. 10.1038/s41598-024-61831-8. 38755300 PMC11098811

[ece370921-bib-0029] Lefort, K. J. , N. E. Hussey , J. M. Jones , K. F. Johnson , and S. H. Ferguson . 2022. “Satellite‐Tracked Sperm Whale Migrates From the Canadian Arctic to the Subtropical Western North Atlantic.” Marine Mammal Science 38: 1242–1248. 10.1111/mms.12909.

[ece370921-bib-0030] Lydersen, C. , J. Vacquié‐Garcia , M. P. Heide‐Jørgensen , N. Øien , C. Guinet , and K. M. Kovacs . 2020. “Autumn Movements of Fin Whales ( *Balaenoptera physalus* ) From Svalbard, Norway, Revealed by Satellite Tracking.” Scientific Reports 10: 16966. 10.1038/s41598-020-73996-z. 33046805 PMC7550606

[ece370921-bib-0031] MacArthur, R. H. 1972. Geographical Ecology: Patterns in the Distribution of Species. Princeton, NJ: Princeton University Press.

[ece370921-bib-0032] Mackintosh, N. A. 1965. The Stocks of Whales. London, UK: Fishing News (Books).

[ece370921-bib-0033] Mate, B. R. , V. Y. Ilyashenko , A. L. Bradford , et al. 2015. “Critically Endangered Western Gray Whales Migrate to the Eastern North Pacific.” Biology Letters 11: 20150071. 10.1098/rsbl.2015.0071. 25878049 PMC4424619

[ece370921-bib-0034] Matthews, C. J. D. , S. P. Luque , S. D. Petersen , R. D. Andrews , and S. H. Ferguson . 2011. “Satellite Tracking of a Killer Whale ( *Orcinus orca* ) in the Eastern Canadian Arctic Documents Ice Avoidance and Rapid, Long‐Distance Movement Into the North Atlantic.” Polar Biology 34: 1091–1096. 10.1007/s00300-010-0958-x.

[ece370921-bib-0035] Pante, E. , B. Simon‐Bouhet , and J. Irisson . 2023. “marmap: Import, Plot and Analyze Bathymetric and Topographic Data. R package Version 1.0.10.” https://CRAN.R‐project.org/package=marmap.

[ece370921-bib-0036] Pitman, R. L. , J. W. Durban , T. Joyce , H. Fearnbach , S. Panigada , and G. Lauriano . 2020. “Skin in the Game: Epidermal Molt as a Driver of Long‐Distance Migration in Whales.” Marine Mammal Science 36: 565–594. 10.1111/mms.12661.

[ece370921-bib-0037] R Core Team . 2023. R: A Language and Environment for Statistical Computing. Vienna, Austria: R Foundation for Statistical Computing. https://www.R‐project.org/.

[ece370921-bib-0038] Risch, D. , M. Castellote , C. W. Clark , et al. 2014. “Seasonal Migrations of North Atlantic Minke Whales: Novel Insights From Large‐Scale Passive Acoustic Monitoring Networks.” Movement Ecology 2: 24. 10.1186/s40462-014-0024-3. 25709833 PMC4337769

[ece370921-bib-0039] Romagosa, M. , M. Baumgartner , I. Cascão , et al. 2020. “Baleen Whale Acoustic Presence and Behaviour at a Mid‐Atlantic Migratory Habitat, the Azores Archipelago.” Scientific Reports 10: 4766. 10.1038/s41598-020-61849-8. 32179826 PMC7075977

[ece370921-bib-0040] Servidio, A. , E. Pérez‐Gil , M. Pérez‐Gil , A. Cañadas , P. S. Hammond , and V. Martín . 2019. “Site Fidelity and Movement Patterns of Short‐Finned Pilot Whales Within the Canary Islands: Evidence for Resident and Transient Populations.” Aquatic Conservation: Marine and Freshwater Ecosystems 29: 227–241. 10.1002/aqc.3135.

[ece370921-bib-0041] Shabangu, F. W. , K. P. Findlay , D. Yemane , et al. 2019. “Seasonal Occurrence and Diel Calling Behaviour of Antarctic Blue Whales and Fin Whales in Relation to Environmental Conditions off the West Coast of South Africa.” Journal of Marine Systems 190: 25–39. 10.1016/j.jmarsys.2018.11.002.

[ece370921-bib-0042] Silva, M. A. , R. Prieto , I. Cascão , et al. 2013. “Spatial and Temporal Distribution of Cetaceans in the Mid‐Atlantic Waters Around the Azores.” Marine Biology Research 10: 123–137. 10.1080/17451000.2013.793814.

[ece370921-bib-0043] Simmonds, M. , and L. Lopez‐Jurado . 1991. “Whales and the Military.” Nature 351: 448. 10.1038/351448a0.

[ece370921-bib-0044] Stern, S. J. , and A. S. Friedlaender . 2018. “Migration and Movement.” In Encyclopedia of Marine Mammals, edited by B. Würsig , J. G. M. Thewissen , and K. Kovacs , 602–606. San Diego, CA: Academic Press.

[ece370921-bib-0045] Stone, G. , L. Florez‐Gonzalez , and S. Katona . 1990. “Whale Migration Record.” Nature 346: 705. 10.1038/346705a0.

[ece370921-bib-0046] Storrie, L. , L. L. Loseto , E. L. Sutherland , S. A. MacPhee , G. O'Corry‐Crowe , and N. E. Hussey . 2023. “Do Beluga Whales Truly Migrate? Testing a Key Trait of the Classical Migration Syndrome.” Movement Ecology 11: 53. 10.1186/s40462-023-00416-y. 37649126 PMC10469428

[ece370921-bib-0047] Szesciorka, A. R. , L. T. Ballance , A. Širović , et al. 2020. “Timing Is Everything: Drivers of Interannual Variability in Blue Whale Migration.” Scientific Reports 10: 7710. 10.1038/s41598-020-64855-y. 32382054 PMC7206123

[ece370921-bib-0048] Van Cise, A. M. , P. R. Wade , C. E. C. Goertz , et al. 2020. “Skin Microbiome of Beluga Whales: Spatial, Temporal, and Health‐Related Dynamics.” Animal Microbiome 2: 39. 10.1186/s42523-020-00057-1. 33499987 PMC7807513

[ece370921-bib-0049] Visser, F. , K. L. Hartman , G. J. Pierce , V. D. Valavanis , and J. Huisman . 2011. “Timing of Migratory Baleen Whales at the Azores in Relation to the North Atlantic Spring Bloom.” Marine Ecology Progress Series 440: 267–279. 10.3354/meps09349.

[ece370921-bib-0050] Whitehead, H. , A. Faucher , S. Gowans , and S. McCarrey . 1997. “Status of the Northern Bottlenose Whale, *Hyperoodon ampullatus*, in the Gully, Nova Scotia.” Canadian Field‐Naturalist 111: 287–292. 10.5962/p.358166.

[ece370921-bib-0051] Whitehead, H. , and S. K. Hooker . 2012. “Uncertain Status of the Northern Bottlenose Whale *Hyperoodon ampullatus* : Population Fragmentation, Legacy of Whaling and Current Threats.” Endangered Species Research 19: 47–61. 10.3354/esr00458.

[ece370921-bib-0052] Whitehead, H. , R. Reeves , L. Feyrer , and R. L. Brownell Jr. . 2021. “ *Hyperoodon ampullatus* . The IUCN Red List of Threatened Species.” e.T10707A50357742. 10.2305/IUCN.UK.2021-1.RLTS.T10707A50357742.en.

[ece370921-bib-0053] Wickham, H. , M. Averick , J. Bryan , et al. 2019. “Welcome to the Tidyverse.” Journal of Open Source Software 4: 1686. 10.21105/joss.01686.

