# Correction to “Aseasonal Migration of a Northern Bottlenose Whale Provides Support for the Skin Molt Migration Hypothesis”

**DOI:** 10.1002/ece3.73135

**Published:** 2026-02-19

Lefort, K. J., L. Storrie, N. E. Hussey, and S. H. Ferguson. 2025. “Aseasonal Migration of a Northern Bottlenose Whale Provides Support for the Skin Molt Migration Hypothesis.” *Ecology and Evolution* 15: e70921. https://doi.org/10.1002/ece3.70921.

All tag‐recorded depth values reported in the original article were incorrect. First, all depth measurements should be doubled. Wildlife Computers tags equipped with extended‐range depth sensors (0–2000 m) report depths at half their actual values unless the tag programming file is uploaded to the Wildlife Computers data portal; this file had not been uploaded when the original manuscript was prepared. Second, independent of this scaling issue, the maximum tag‐recorded depth value reported in the original article was incorrectly transcribed. The corrected text, including the updated values, and the revised Figure 2 with its caption are provided below:

**Results (first paragraph):**

“The maximum tag‐recorded depth was 1904 m.”

**Results (second paragraph):**

“Daily maximum tag‐recorded depths were deep during both the foraging (864.4 ± 249.5 m) and directed‐movement (1064.7 ± 386.6 m) phases. Furthermore, maximum daily depths ≥ 200 m were recorded on 52 of 55 days with available data during the foraging and directed‐movement phases. During the proposed molting phase, the whale occupied warmer SSTs (23.2°C ± 0.4°C; tag‐recorded temperature = 23.1°C ± 0.3°C) and shifted its dive behavior to remain near the ocean's surface (daily maximum tag‐recorded depth = 59.0 ± 106.3 m). This included six consecutive days (July 30–August 4) where the tag‐recorded depth did not exceed 23 m.”

**Discussion (third paragraph):**

“Secondly, the tagged whale, which is a known deep‐water forager (Hooker and Baird 1999), was recorded only reaching a maximum depth of 23 m during the suspected molt (with the exception of one dive to 300 m on the last day of the suspected molt).”

“This is in contrast to the whale's dive behavior on the Arctic foraging grounds and during its directed‐movement phase, where frequent deep dives (> 1000 m) were recorded, perhaps allowing the whale to feed in preparation for and to recover from a week‐long period of fasting during the molt.”

“Nonetheless, the 6‐day hiatus in dives beyond 23 m at the southern terminus of the whale's movement is strong evidence that this migration was not to feed.”

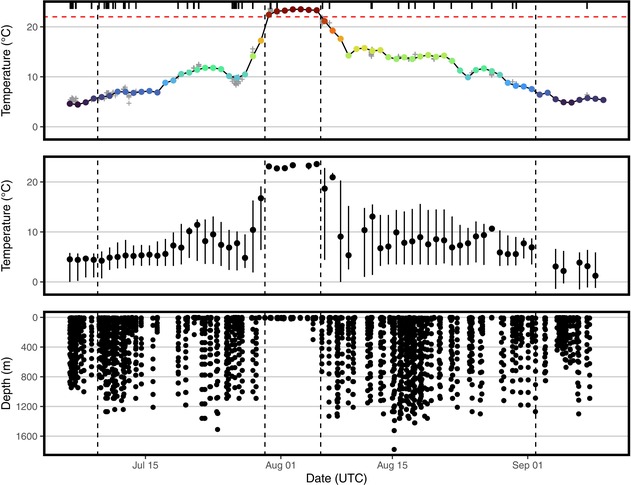

**FIGURE 2**. Top: Mean daily sea‐surface temperature (°C; color‐coded by temperature to match Figure 1) along the northern bottlenose whale's (*Hyperoodon ampullatus*) migration (obtained from the OISST database). Gray crosses show tag‐recorded temperature data when at the ocean's surface (< 10 m depth), demonstrating agreement between tag‐recorded temperature and the OISST. Rug plot shows the temporal distribution of tag‐recorded temperature data when at the ocean's surface. Horizontal red dashed line shows 22.0°C, the temperature threshold used to define the molting phase. Vertical dashed lines show the breaks between the three hypothetical movement phases: foraging phase (July 5–8 and September 2–10), directed‐movement phase (July 9–29 and August 6–September 1), and molting phase (July 30–August 5). Middle: Tag‐recorded temperature (°C) along the whale's migration. Points show daily means. Vertical bars show daily minima and maxima. Bottom: Tag‐recorded depth (m) along the whale's migration.

The Supporting Information in the online version of the article has been corrected accordingly.

The above corrections do not affect the published article's conclusions.

We apologize for these errors.

