# Supplementary figures and images for: Aseasonal Migration of a Northern Bottlenose Whale Provides Support for the Skin Molt Migration Hypothesis
Source: Ecol Evol. 2025 Jan 30;15(2):e70921. doi: 10.1002/ece3.70921 (PMC11780396; doi:10.1002/ece3.70921)

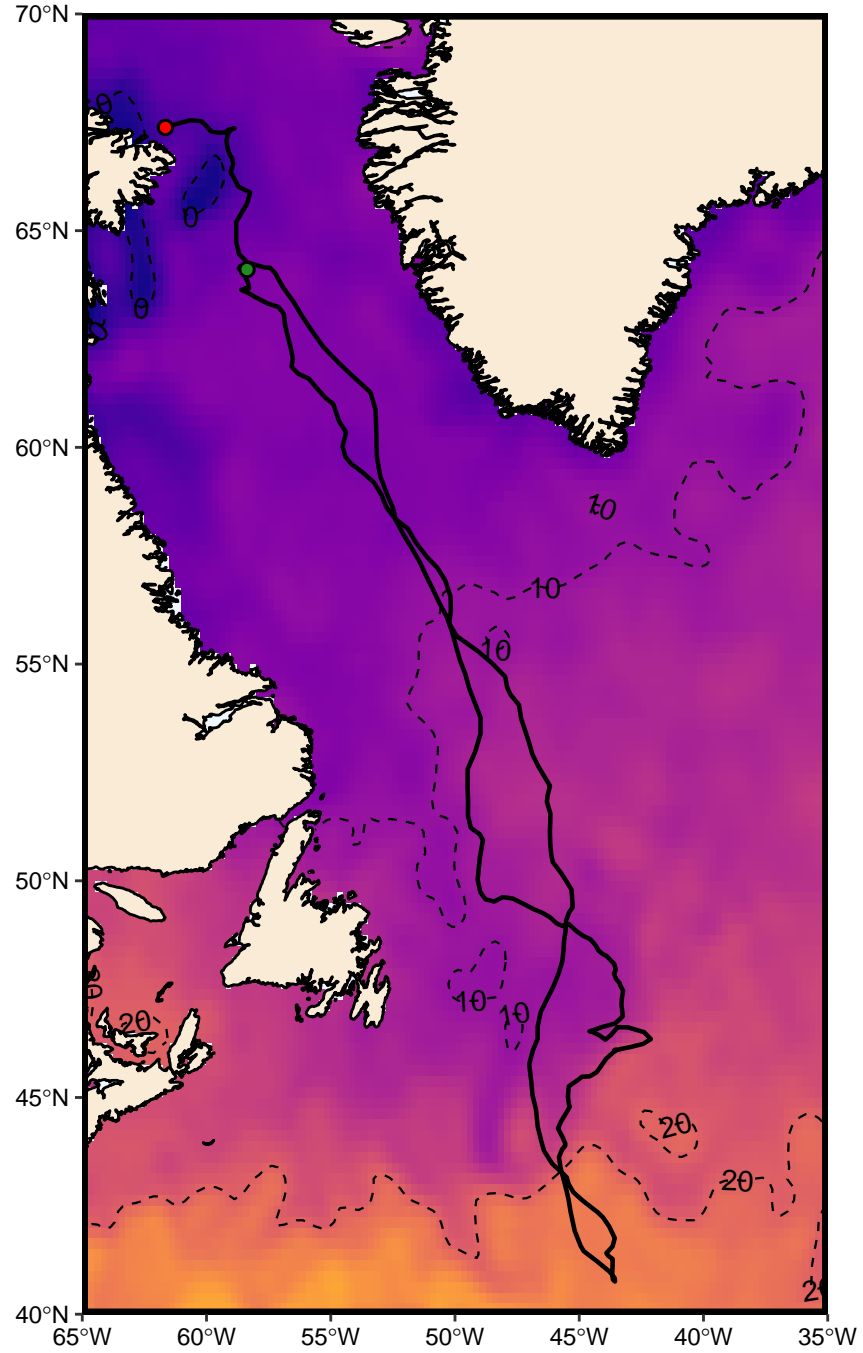

Supplement: Supplementary file 6 — Figure S1. Model‐estimated path of an adult male northern bottlenose whale ( Hyperoodon ampullatus ) satellite tracked along the eastern Canadian coast from July 5 to September 10, 2019. The green circle is the tagging location and the red circle is the final model‐estimated location. The ocean is color‐coded by sea‐surface temperature (°C) on July 30, 2019 (the first day of the proposed molt) obtained from the OISST database. Isotherms are drawn at 0°C, 10°C, and 20°C (dashed lines). [file ECE3-15-e70921-s005.pdf]

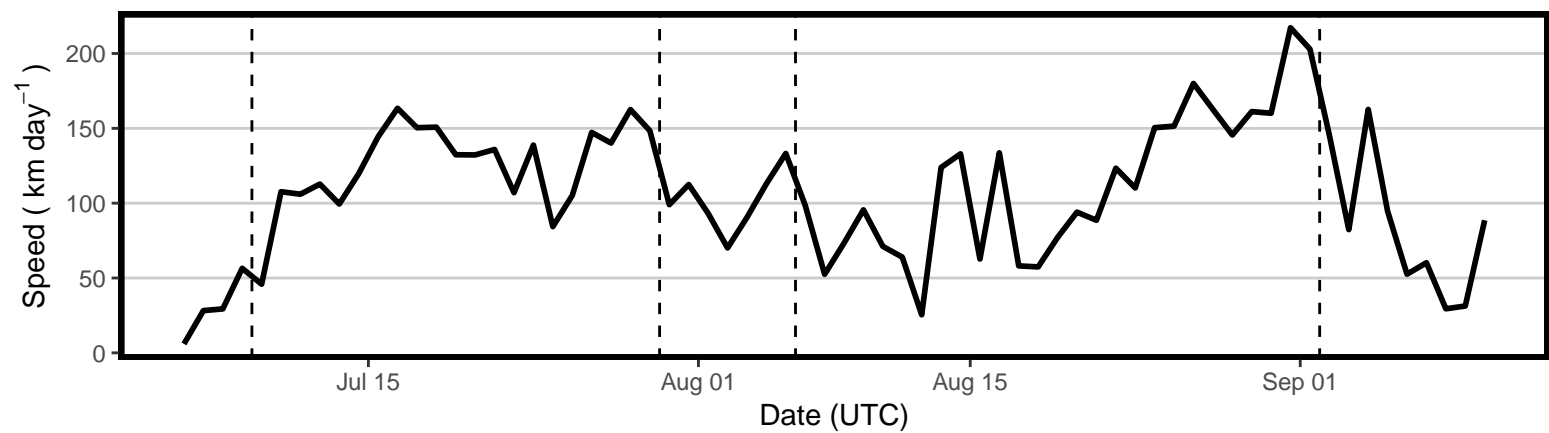

Supplement: Supplementary file 7 — Figure S2. Daily distance traveled (km day−1) along the northern bottlenose whale's ( Hyperoodon ampullatus ) satellite‐tracked path. Vertical dashed lines show the breaks between the three hypothetical movement phases: foraging phase (July 5–8 and September 2–10), directed‐movement phase (July 9–29 and August 6–September 1), and molting phase (July 30–August 5). [file ECE3-15-e70921-s003.pdf]

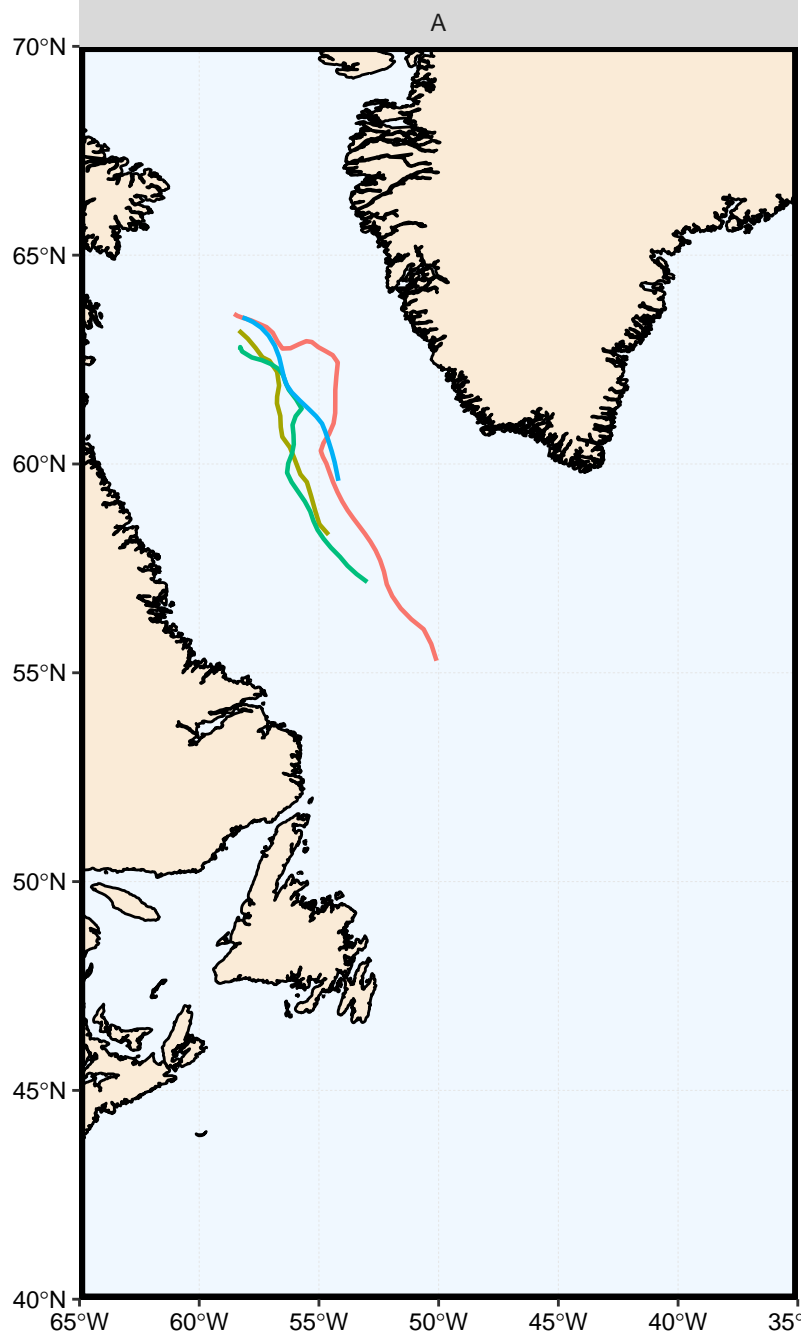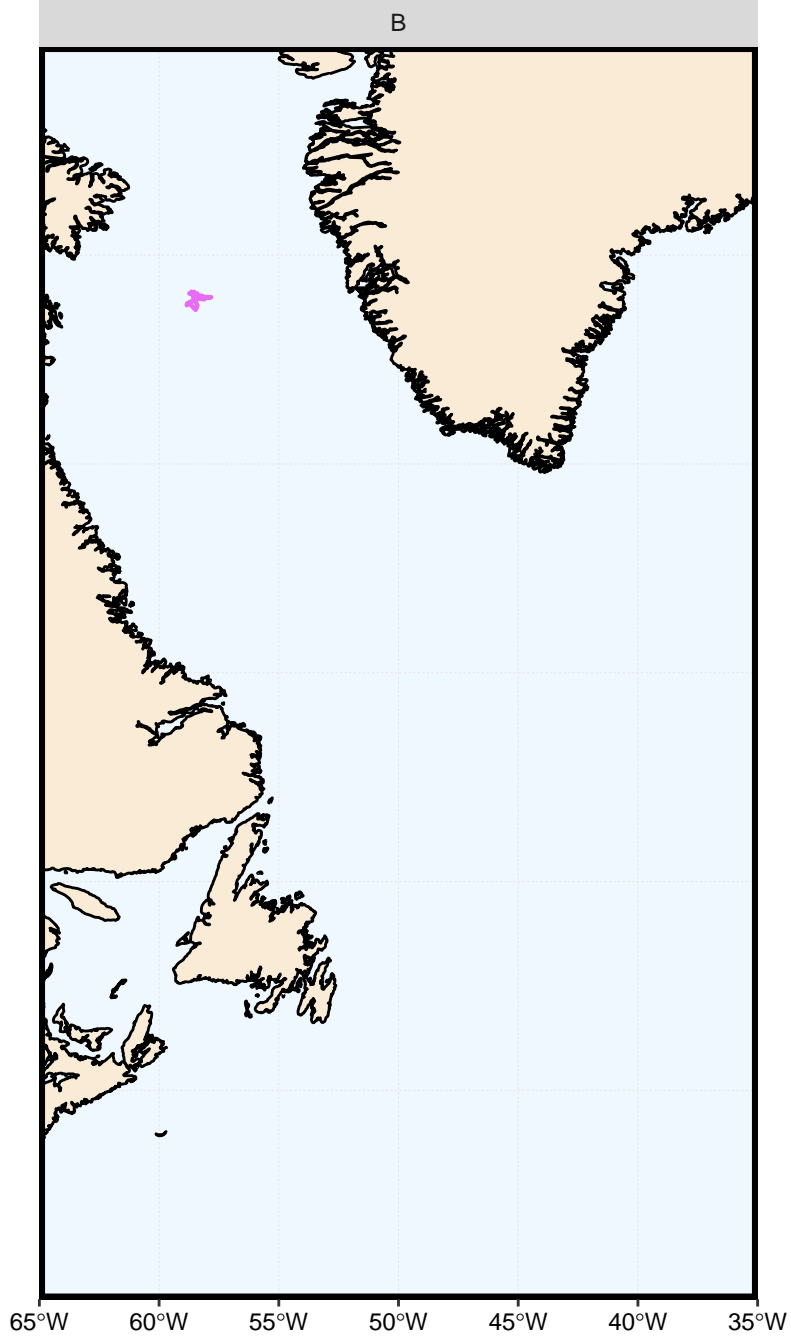

Supplement: Supplementary file 8 — Figure S3. Model‐estimated path of five other adult northern bottlenose whales ( Hyperoodon ampullatus ) satellite‐tagged in Davis Strait in July 2019. Panel A: four whales which initiated southward movements (but whose transmissions were not sustained long enough to document a complete round‐trip migration, if one did occur). Panel B: one whale which did not initiate a southward movement. Each color represents an individual whale. [file ECE3-15-e70921-s006.pdf]

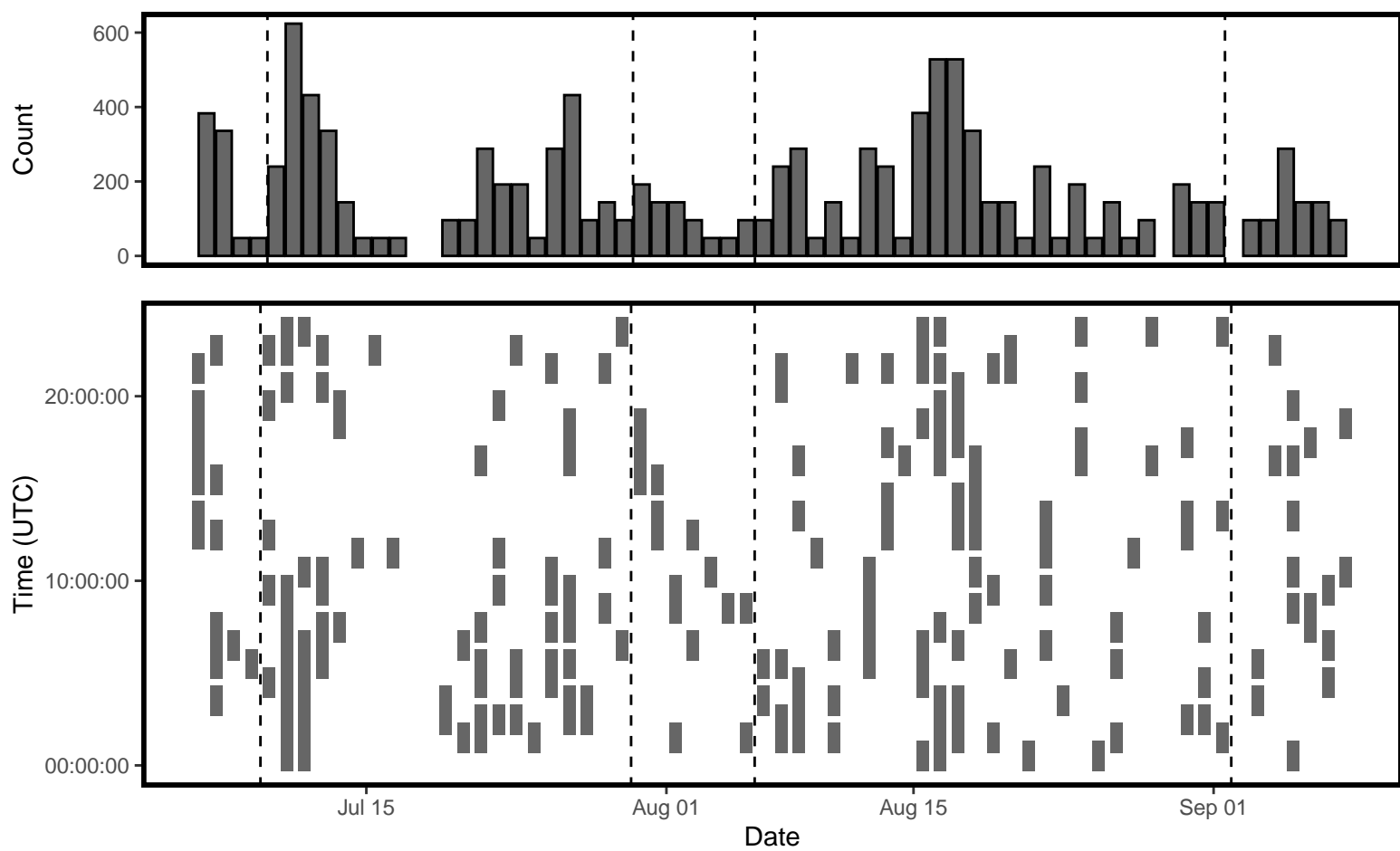

Supplement: Supplementary file 9 — Figure S4. Top: Histogram showing the number of tag‐recorded depth observations collected each day from a satellite‐tracked northern bottlenose whale ( Hyperoodon ampullatus ). Bottom: Diel distribution of tag‐recorded depth observations collected each day. Vertical dashed lines show the breaks between the three hypothetical movement phases: foraging phase (July 5–8 and September 2–10), directed‐movement phase (July 9–29 and August 6–September 1), and molting phase (July 30–August 5). [file ECE3-15-e70921-s009.pdf]

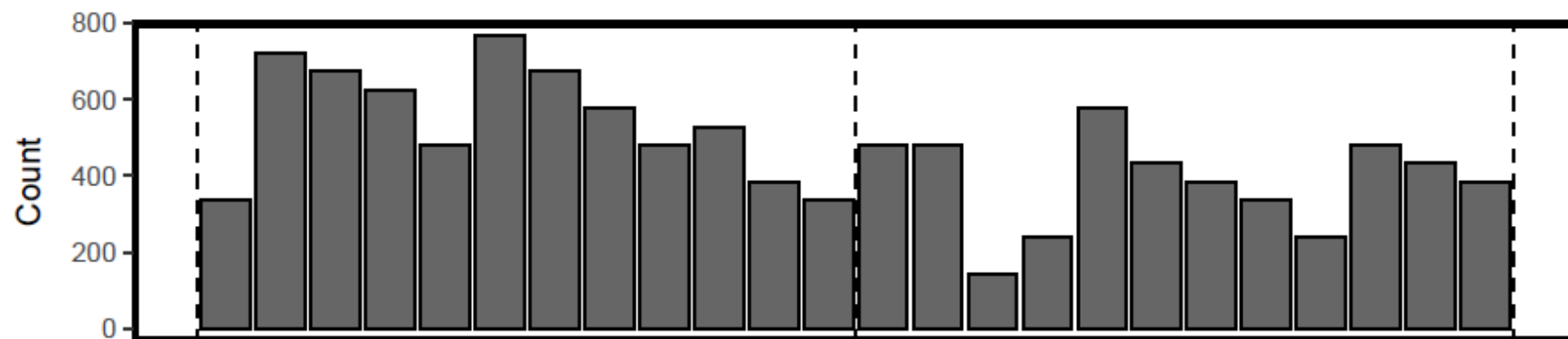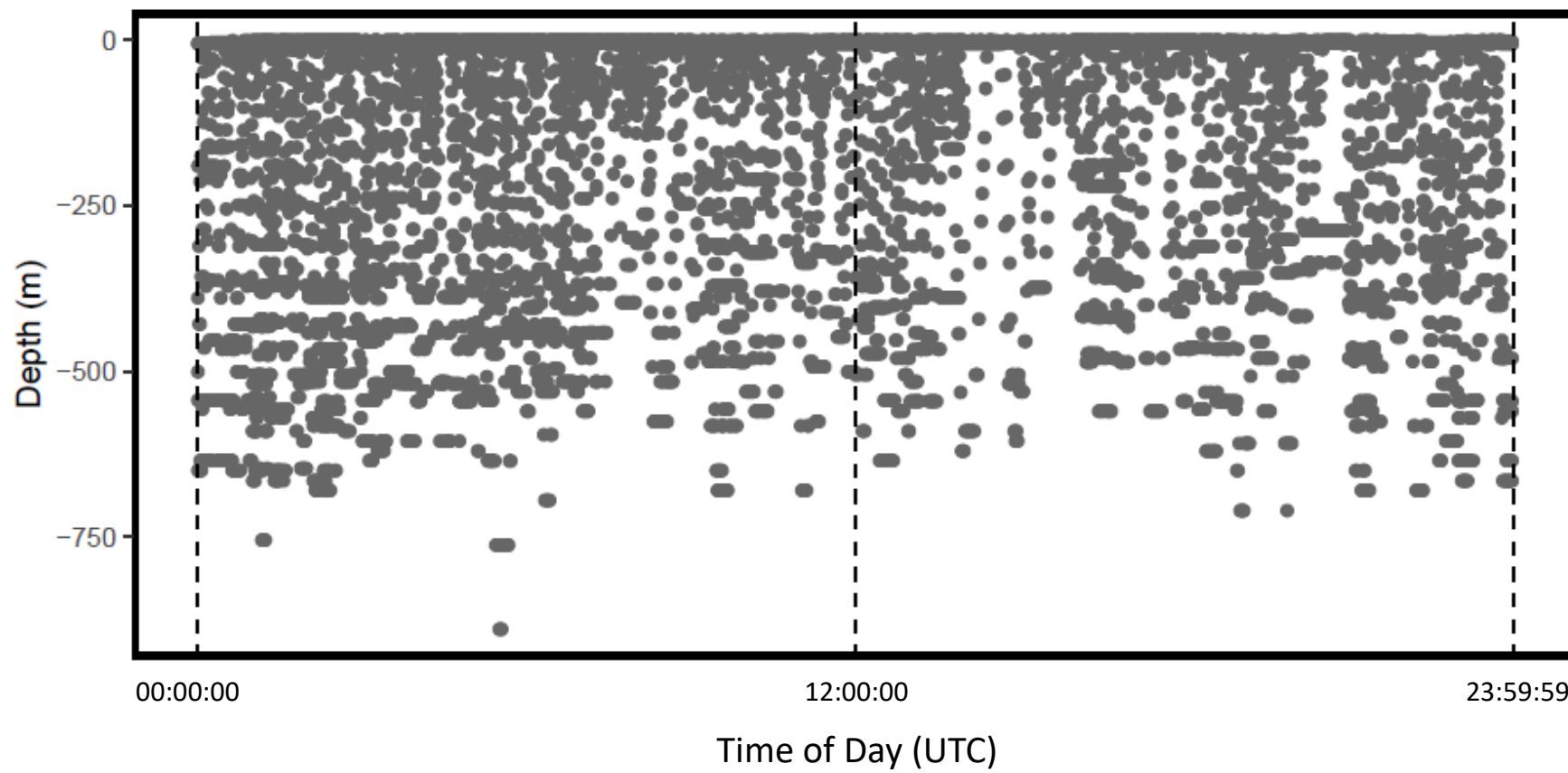

Supplement: Supplementary file 10 — Figure S5. Top: Histogram showing the number of tag‐recorded depth observations binned by hour of day from a satellite‐tracked northern bottlenose whale ( Hyperoodon ampullatus ). Bottom: raw tag‐recorded depth (m) by time of day (UTC) across the entire deployment. The whale occupied longitudes between 42° W and 62° W, occupying time zones −3 and −4 UTC; thus, midday local time ranged from 09:00 to 08:00 UTC. [file ECE3-15-e70921-s002.pdf]
